# Supplementary material for: c-FLIP is crucial for IL-7/IL-15-dependent NKp46+ ILC development and protection from intestinal inflammation in mice
Source: Nat Commun. 2020 Feb 26;11:1056. doi: 10.1038/s41467-020-14782-3 (PMC7044440; doi:10.1038/s41467-020-14782-3)
Supplement: Supplementary file 1 — Supplementary Information [file 41467_2020_14782_MOESM1_ESM.pdf]

SUPPLEMENTARY INFORMATION

c-FLIP is crucial for IL-7/IL-15-dependent NKp46$^+$ ILC development and protection from intestinal inflammation in mice

By Bank et al.

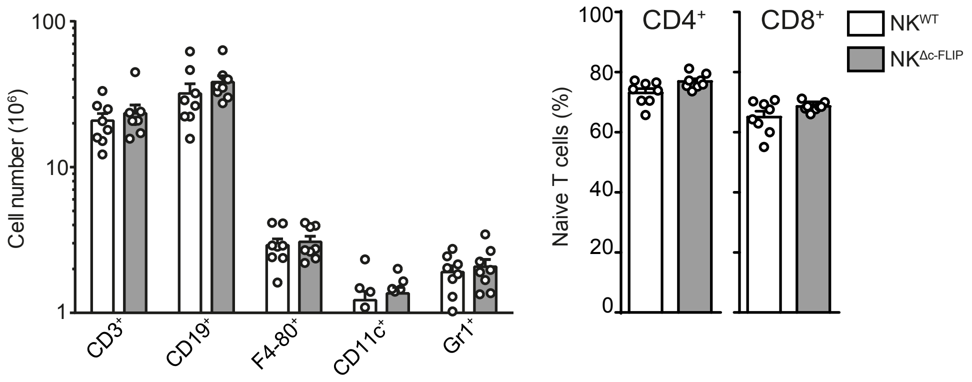

Supplementary Figure 1. Numbers of splenic CD3⁺ T and CD19⁺ B lymphocytes, F4-80⁺ Macrophages, CD11c⁺ dendritic cells and Gr-1⁺ granulocytes (left) and frequencies of naive $CD44^{lo}CD62L^{hi}$ CD4⁺ and CD8⁺ T cells (right) are not altered in $NK^{\Delta c\text{-}FLIP}$ mice. Data obtained from 8 mice per group were pooled and are shown as mean +SEM (one experiment). Statistical significances were determined using two-tailed Mann–Whitney U test. Source data are provided as a source data file.

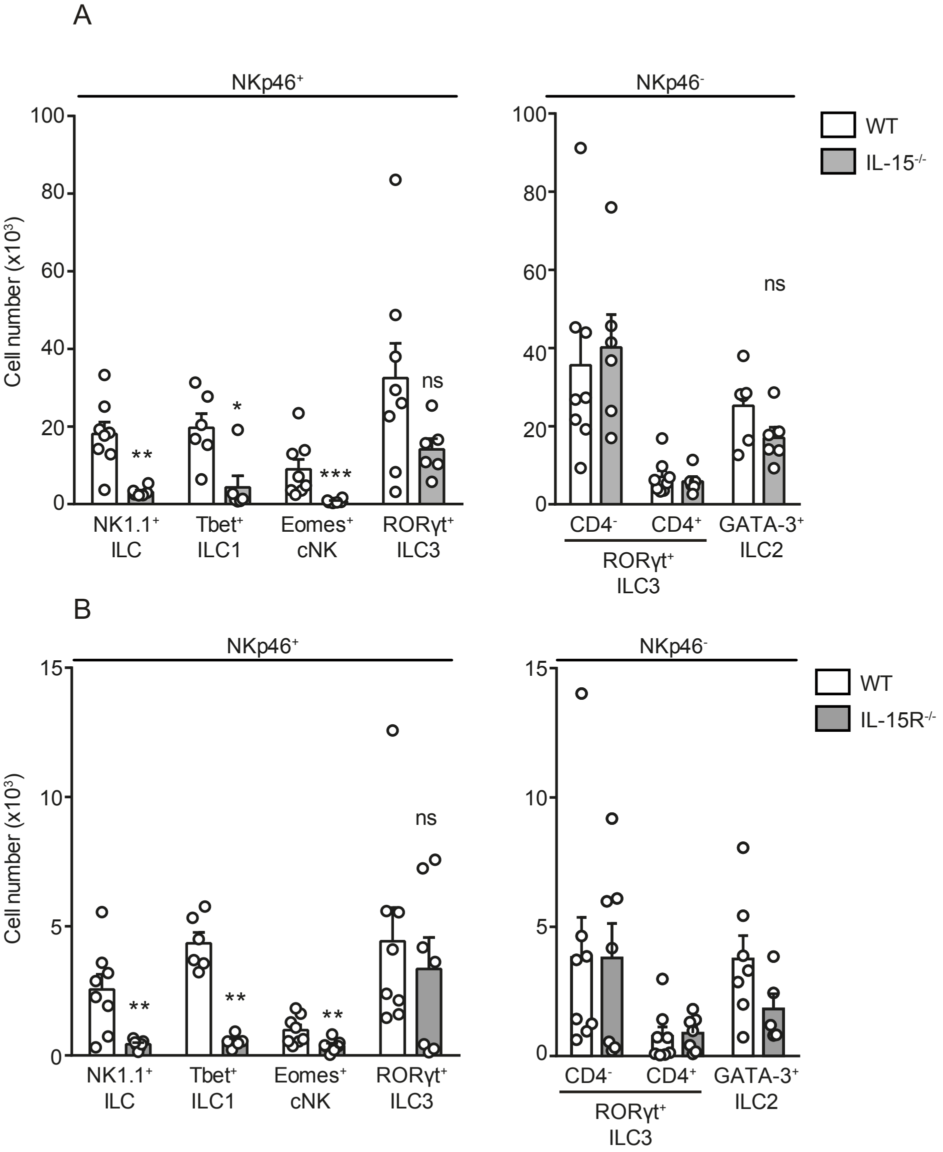

Supplementary Figure 2. IL-15 promotes the maintenance of small intestinal lamina propria ILC1s and cNKs. Leukocytes were isolated from the small intestinal lamina propria of **(A)** IL-15$^{-/-}$, **(B)** IL15Rα$^{-/-}$ mice and appropriate WT controls. Cells were analyzed by flow cytometry as described in Fig. 3. Shown are pooled results (means +SEM) from **(A)** 6 experiments with a total of 6-8 mice per genotype and **(B)** 5 experiments with a total of 6-8 mice. **(A, B)** For NKp46^{+} and NKp46^{-} ILC **(A)** 6-8 and **(B)** 5-8 samples were analyzed, respectively. Statistical significances were determined using two-tailed Mann–Whitney U test (* $p \leq 0.05$; ** $p \leq 0.005$; *** $p \leq 0.001$; ns = not significant). Source data are provided as a source data file.

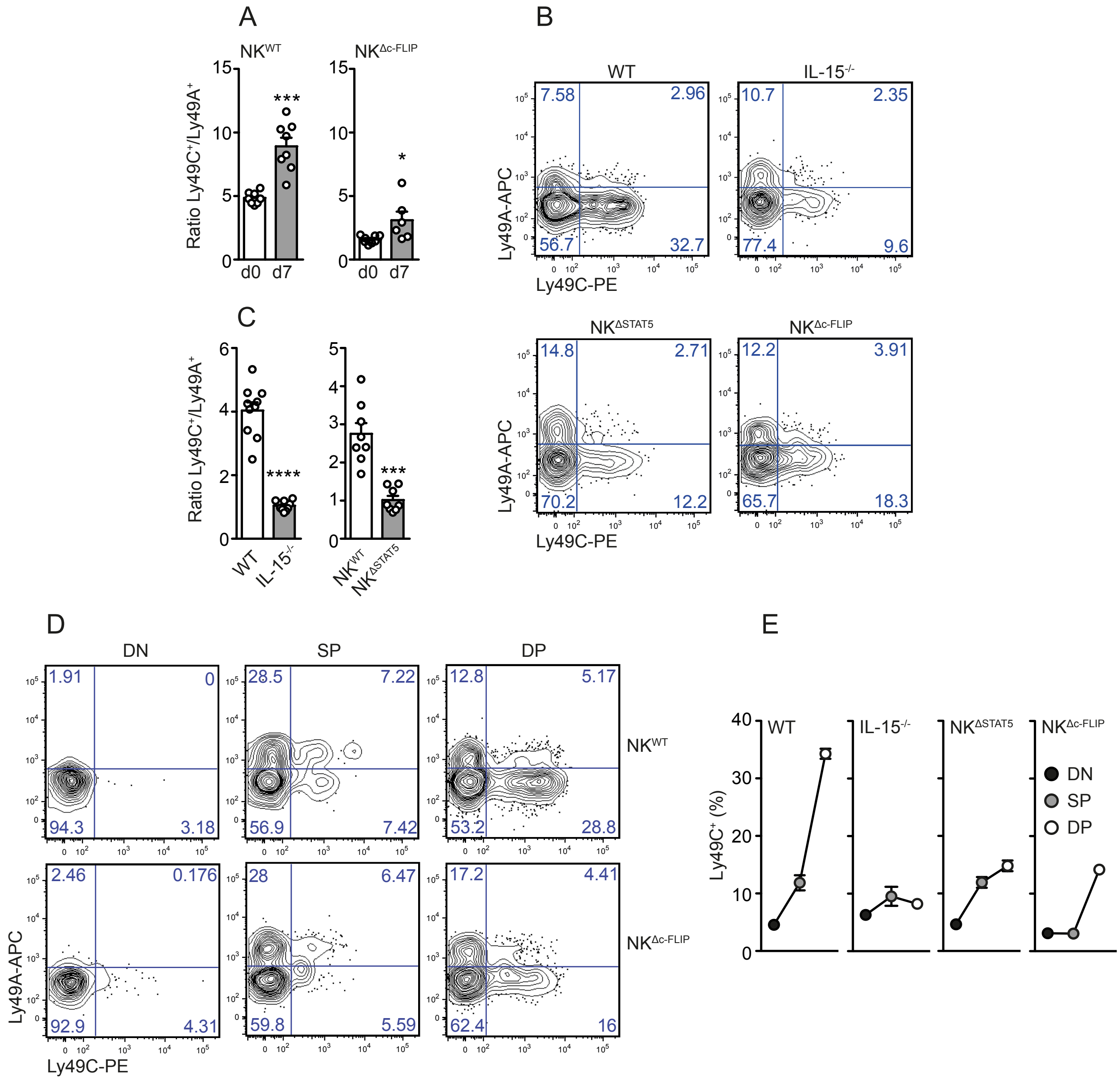

Supplementary Figure 3. c-FLIP is required for the generation and disease-related accumulation of Ly49C⁺ cNK. (A-C) Spleen- and **(D, E)** bone marrow (BM)-derived Lin⁻CD122⁺ NK1.1⁺NKp46⁺ cNK of the indicated mouse strains were analyzed by flow cytometry to determine the frequencies of Ly49A⁺ and Ly49C⁺ cells. **(A)** Shown are Ly49C⁺/Ly49A⁺ ratios (mean + SEM) of splenic cNK from untreated (d0) and DSS-treated (d7) NK^{WT} and $NK^{\Delta c\text{-}FLIP}$ mice (acute colitis; see Fig. 4). **(B)** Representative contour plots and **(C)** Ly49C⁺/Ly49A⁺ ratios (mean + SEM) are shown for a total of 8-10 untreated mice per genotype (2-3 independent experiments). Numbers indicate percentages. **(D, E)** BM Lin⁻CD122⁺ NK1.1⁻NKp46⁻ (DN), NK1.1⁺NKp46⁻ (SP) and NK1.1⁺NKp46⁺ (DP) cells of the indicated mouse strains were analyzed for Ly49A and Ly49C expression. Shown are **(D)** representative contour plots and **(E)** pooled results (mean ± SEM) from 1-3 independent experiments with a total of 8-10 mice per group. **(D)** Numbers indicate percentages. **(A)** 6-8, **(C)** 8-10 and **(E)** 8-10 tissue samples were analyzed. Statistical significances were determined using two-tailed Mann–Whitney U or paired Student's t test (* $p \le 0.05$; ** $p \le 0.005$; *** $p \le 0.001$; **** $p \le 0.0001$). **(A, E)** Source data are provided as a source data file.

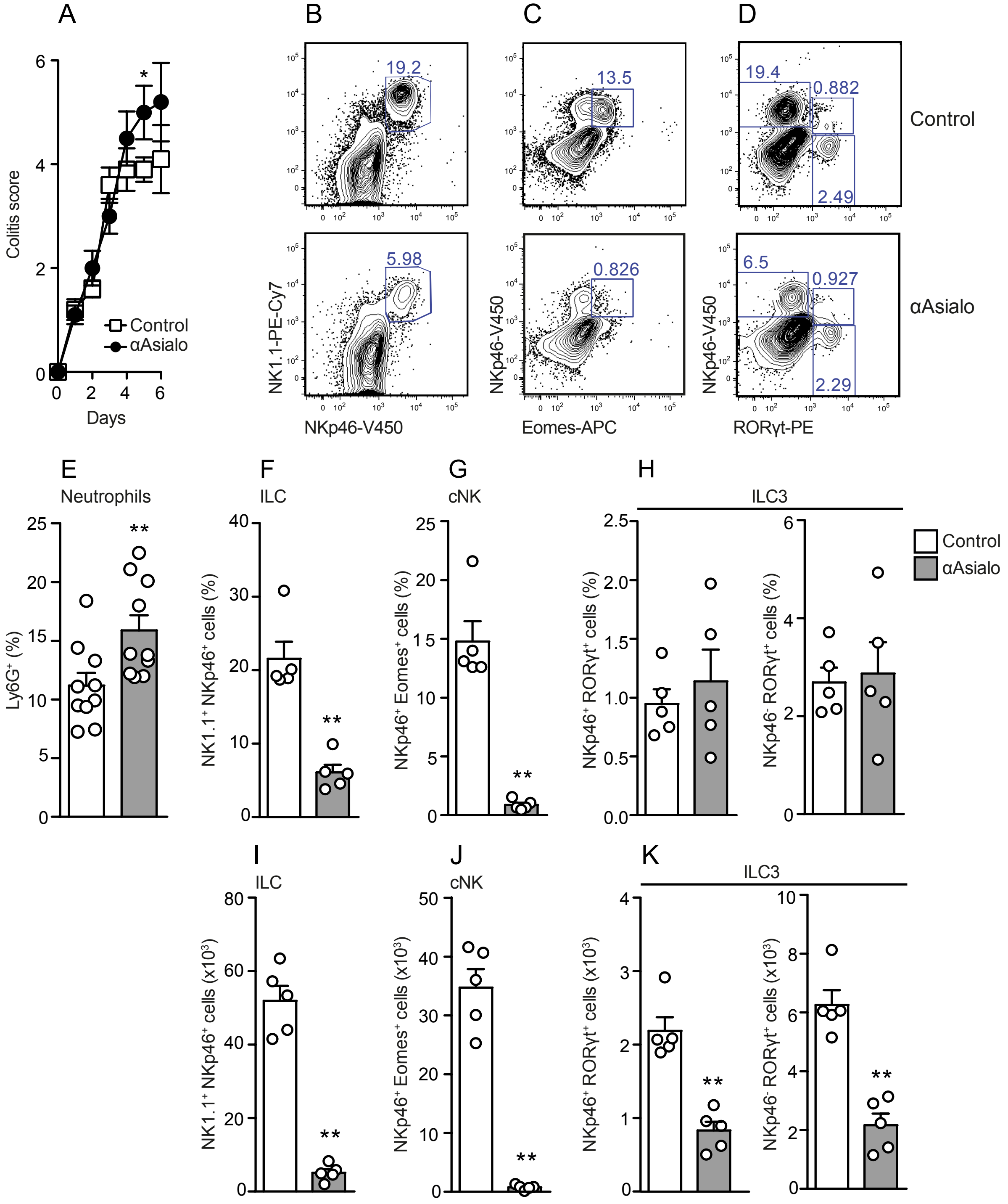

Supplementary Figure 4. Antibody-mediated depletion of cNK aggravates acute intestinal inflammation. (A-K) C57BL/6 mice were injected four times i.p. with cNK-depleting anti-Asialo-GM1 (αAsialo) or unspecific control antibody (Control). Two days after the second injection, a 5-day treatment with DSS was initiated. Shown are data of one representative experiment out of two (each experiment with 10 mice per group). **(A)** Colitis scores (mean ± SEM) were determined on a daily basis. To define the frequencies **(B-D, F-H)** and absolute numbers **(I-K)** of the indicated ILC subsets, colonic lamina propria cells of two mice were pooled at day 7. Lin^-CD45^+ cells were analyzed by flow cytometry (5 samples per group). **(E)** The frequencies of $CD3^-Ly6G^+$ leukocytes were determined in peripheral blood of 10 individual mice at day 7. **(E-K)** Data show mean + SEM. Statistical significances were determined using two-tailed Mann–Whitney U test (* p ≤ 0.05; **p ≤0.005). **(A, E-K)** Source data are provided as a source data file.

Antibody	Fluorochrome	Clone	Company	Order No	Dilution
CD3	FITC	145-2C11	BD Biosciences	561826	200
CD3	BV421	145-2C11	Biolegend	100336	200
CD3	BV510	17A2	Biolegend	100233	200
CD4	FITC	RM4-5	eBioscience / Biolegend	11-042-81 / 100510	300
CD4	PerCPCy5.5	RM4-5	Biolegend	105540	300
CD4	APC	RM4-5	Biolegend	100412	300
CD4	BV510	RM4-5	Biolegend	100559	400
CD4	FITC	RM4-5	Biolegend	100510	400
CD5	BV510	53-7.3	Biolegend	100627	400
CD8a	a488	53-6.7	Biolegend	100723	200
CD8a	APC eF780	53-6.7	eBioscience	47-0081-82	300
CD8a	BV510	53-6.7	Biolegend	100751	400
CD8a	FITC	53-6.7	BD Biosciences	553030	200
CD11b	BV510	M1/70	Biolegend	100245	200
CD11b	PE	M1/70	BD Biosciences	557397	200
CD11b	APC-Cy7	M1/70	Biolegend	101225	200
CD11b	APC-eF780	M1/70	eBiosciences	47-0112-82	201
CD11b	a488	M1/70	eBosciences	53-0112-82	200
CD11b	BV510	M1/70	Biolegend	101245	100
CD11c	BV510	N418	Biolegend	117338	200
CD16/31	unlabeled	2.4G2 (ATCC HB197	culture supernatant (1.2 mg/ml)		1000
CD16/32	unlabeled	93	eBioscience	14-0161-82	50
CD19	BV510	6D5	Biolegend	101245	200
CD19	biotin	6D5	Biolegend	115503	200
CD19	FITC	eBio1D3	eBiosciences	E00188-1631	100
CD45.2	BV510	104	Biolegend	109837	333
CD45	APC-Cy7	30-F11	Biolegend	109818	100
CD45.2	A647	104	Biolegend	109824	300
CD90.2	FITC	53-2.1	Biolegend	140304	400
CD122	PE	TM-β1	Biolegend	123210	200
CD122	PE-Cy7	TM-β1	Biolegend	123216	200
CD122	FITC	5H4	BD Biosciences	554452	200
c-Flip	APC	polyclonal	Abcam	ab8421	200
Eomes	APC	REA116	Miltenyi Biotec	130-102-378	15
Eomes	a488	Dan11mag	eBiosciences	53-4875-82	100
FAS (CD95)	purified unlabeled	3C82	Enzo	ALX-804-273	10µg/ml
F4/80	PE-Cy7	BM8	Biolegend	123113	333
GATA3	PE	TWAJ	eBiosciences	12-9966	100
GM-CSF	PE	MP1-22Ep	Biolegend	505405	100
Gr-1	FITC	RB6-8C5	Biolegend	108405	1000
Gr-1	BV510	RB6-8C5	Biolegend	108437	1000
Gr-1	biotin	RB6-8C5	Biolegend	108403	300
Ly49A	APC	A1	Miltenyi Biotec	130-102-371	10
Ly49C	PE	REA253	Miltenyi Biotec	130-102-754	10
Ly6C	PE-Cy7	HK1.4	Biolegend	128017	300
Ly6C	PerCP-Cy5.5	HK1.4	eBioscience	45-5932-82	333
Ly6C	FITC	HK1.4	Biolegend	128005	200
Ly6G	PE	1A8	BD Pharmingen	561104	200
Ly6G	biotin	1A8	Biolegend	127603	300
Ly6G	BV421	1A8	Biolegend	127627	200
Ly6G	BV510	1A8	Biolegend	127633	400
MCP-1	APC	2H5	Biolegend	505909	200
NK1.1	APC-Cy7	PK136	Biolegend	108723	100
Nk1.1	PE-Cy7	PK136	Biolegend	108714	100
NK1.1	BV421	PK136	Biolegend	B170162	200
NKp46	PE-Cy7	29A13	Biolegend	B180585	200
NKp46	V450	29A14	BD Biosciences	500763	75-100
NKp46	PE-Cy7	29A14	Biolegend	137617	200
NKp46	eFluor 660	29A14	eBioscience	50-3351-82	200
pSTAT5 (pY694)	PE	47/Stat5	BD Biosciences	612567	10
SA	FITC		BD Biosciences	554060	300
RORgt	PE	B2D	BD Biosciences	126981-82	100
TER119	FITC	TER-119	Biolegend	116205	300
Ter119	biotin	TER-119	Biolegend	116203	200
Tbet	APC	4B10	Biolegend	664813	100
TNF	purified unlabeled	MP6-XT22	Biolegend	506309	10µg/ml
TRAIL	purified unlabeled	N2B2	Biolegend	109302	10µg/ml
Fixable viability	eF506		eBiosciences	65-0866	1000
Zombie	Violet		Biolegend	423113	1000
7AAD			Biolegend	420403	100

Supplementary Table 1. Antibodies used in this study
