# Supplementary material for: c-FLIP is crucial for IL-7/IL-15-dependent NKp46+ ILC development and protection from intestinal inflammation in mice
Source: Nat Commun. 2020 Feb 26;11:1056. doi: 10.1038/s41467-020-14782-3 (PMC7044440; doi:10.1038/s41467-020-14782-3)
Supplement: Supplementary file 3 — Reporting Summary [file 41467_2020_14782_MOESM3_ESM.pdf]

nature research

Corresponding author(s): Thomas Schüler

Last updated by author(s): Jan 18, 2020

Reporting Summary

Nature Research wishes to improve the reproducibility of the work that we publish. This form provides structure for consistency and transparency in reporting. For further information on Nature Research policies, see Authors & Referees and the Editorial Policy Checklist .

Statistics

For all statistical analyses, confirm that the following items are present in the figure legend, table legend, main text, or Methods section.

n/a	Confirmed	
☐	☒	The exact sample size (n) for each experimental group/condition, given as a discrete number and unit of measurement
☐	☒	A statement on whether measurements were taken from distinct samples or whether the same sample was measured repeatedly
☐	☒	The statistical test(s) used AND whether they are one- or two-sided<br>*Only common tests should be described solely by name; describe more complex techniques in the Methods section.*
☐	☒	A description of all covariates tested
☐	☒	A description of any assumptions or corrections, such as tests of normality and adjustment for multiple comparisons
☒	☐	A full description of the statistical parameters including central tendency (e.g. means) or other basic estimates (e.g. regression coefficient) AND variation (e.g. standard deviation) or associated estimates of uncertainty (e.g. confidence intervals)
☒	☐	For null hypothesis testing, the test statistic (e.g. F, t, r) with confidence intervals, effect sizes, degrees of freedom and P value noted<br>*Give P values as exact values whenever suitable.*
☒	☐	For Bayesian analysis, information on the choice of priors and Markov chain Monte Carlo settings
☒	☐	For hierarchical and complex designs, identification of the appropriate level for tests and full reporting of outcomes
☒	☐	Estimates of effect sizes (e.g. Cohen's d, Pearson's r), indicating how they were calculated

Our web collection on statistics for biologists contains articles on many of the points above.

Software and code

Policy information about availability of computer code

Data collection	Usearch8.1 software package (http://www.drive5.com/usearch/)<br>BD FacsDIVA Software version 6.1.3. and 6.2<br>LightCycler® 96 SW 1.1 (Roche)<br>ScanIt RE4.1
Data analysis	UPARSE algorithm<br>FlowJo Version versions 9.9.6 and 10.0.8r1<br>GraphPad Prism 5 and 6.07

For manuscripts utilizing custom algorithms or software that are central to the research but not yet described in published literature, software must be made available to editors/reviewers. We strongly encourage code deposition in a community repository (e.g. GitHub). See the Nature Research guidelines for submitting code & software for further information.

Data

Policy information about availability of data

All manuscripts must include a data availability statement. This statement should provide the following information, where applicable:

- Accession codes, unique identifiers, or web links for publicly available datasets
- A list of figures that have associated raw data
- A description of any restrictions on data availability

16S rRNA gene sequencing data have been deposited in the NCBI (Bioproject Database) under the accession number: PRJNA437582 [https://www.ncbi.nlm.nih.gov/bioproject/?term=PRJNA437582]. The source data underlying Figs 1A-D, 2A-F, 3E-K, 4A-F and I-M, 5A-C and E-H, 6A-F, 7C and D, 8A-F, I and K and Supplementary Figs 1, 2, 3A and E, 4A and E-K are provided as a Source Data file. All other data that support the findings of this study are available from the corresponding author on reasonable request.

Field-specific reporting

Please select the one below that is the best fit for your research. If you are not sure, read the appropriate sections before making your selection.

☒ Life sciences ☐ Behavioural & social sciences ☐ Ecological, evolutionary & environmental sciences

For a reference copy of the document with all sections, see nature.com/documents/nr-reporting-summary-flat.pdf

Life sciences study design

All studies must disclose on these points even when the disclosure is negative.

Sample size	To estimate sample size of the experimental groups in our colitis experiments we used the nQuery Advisor software as well as the following web-based tool http://www.quantitativeskills.com/sisa/calculations/samsize.html<br>Given a type I error rate α of 5% and a desired statistical power of 80% and an expected effect size (Cohen´s d; defined as difference between the means of two data sets, divided by the expected standard deviation) of 1 to 2 (low to medium) in a two-tailed statistical analysis, a minimum sample size of 16 per group was calculated to be necessary. In long-term chronic colitis experiments a minimum sample size of 20 per group was estimated including up to 4 potential drop-out animals. These sample sizes were approved and accepted by the local animal welfare legislations. We usually performed two independent experiments to reach in total this calculated sample size and to test reproducibility the observation.<br>Wherever statistical analysis was performed, total sample sizes (n) are defined (summary of data of several independent experiments) in figure legends. Where error bars are used center values (mean) are defined and p-values are given.
Data exclusions	No data were excluded.
Replication	All experimental findings were reproduced. In many instances, experimental data from several expriments have been summarized (as indicated).
Randomization	No randomization was performed during group allocation. Littermate mice were ear tagged and genotyped in order to determine genetic genotype and to generate experimental groups. Each cohort of littermate mice were co-housed in order to avoid microbiome differences due to separate housing. Animals of similar age and sex (littermates if ever possible) were used to control for covariates.
Blinding	Investigators were not blinded during data collection and analysis.

Reporting for specific materials, systems and methods

We require information from authors about some types of materials, experimental systems and methods used in many studies. Here, indicate whether each material, system or method listed is relevant to your study. If you are not sure if a list item applies to your research, read the appropriate section before selecting a response.

Materials & experimental systems

n/a	Involved in the study	
☐	☒	Antibodies
☐	☒	Eukaryotic cell lines
☒	☐	Palaeontology
☐	☒	Animals and other organisms
☒	☐	Human research participants
☒	☐	Clinical data

Methods

n/a	Involved in the study	
☒	☐	ChIP-seq
☐	☒	Flow cytometry
☒	☐	MRI-based neuroimaging

Antibodies

Antibodies used	Information is provided in Methods and Supplemantary Table 1
Validation	All the antibodies are from commercial sources and have been validated by the vendors. The validation data are available on the manufacturer's website. Antibody with their respective clone number, catalogue number and provider/manufacturer is mentioned below.

Eukaryotic cell lines

Policy information about cell lines

Cell line source(s)	OP9 cells (ATCC® CRL-2749™)
Authentication	The cell line has not been authenticated.
Mycoplasma contamination	OP9 cells were not tested for mycoplasma infection.
Commonly misidentified lines (See ICLAC register)	No commonly misidentified cell lines were used.

Animals and other organisms

Policy information about studies involving animals; ARRIVE guidelines recommended for reporting animal research

Laboratory animals	NKp46iCre<br>c-FLIP fl/fl<br>STAT5 fl/fl<br>IL-15-/-<br>IFN-γ reporter mice (Great)<br>RAG1-/-<br>IL-7R-/-<br>IL-7R fl/fl Jackson Laboratory, stock no. 022143<br>IL-15Rα-/- Jackson Laboratory, stock no. 003723<br>Eomes fl/fl Jackson Laboratory, stock no. 017293<br>C57Bl6 Jackson Laboratory<br><br>All mice were on a C57Bl6 background, except of B6; 129SF2/J (recommend control for IL15Rα-/-);<br>both sexes were used in comparable numbers; age 8-20 weeks.<br><br>Experimental procedures were approved by the relevant animal experimentation committee and performed in compliance with international and local animal welfare legislations (Landesverwaltungsamt Sachsen-Anhalt, AZ 42502-2-1202 and AZ 42502-2-1521 Uni MD).
Wild animals	This study did not involve wild animals.
Field-collected samples	The study did not include samples collected from the field.
Ethics oversight	Landesverwaltungsamt Sachsen-Anhalt (permit numbers AZ 42502-2-1202 and AZ 42502-2-1521 Uni MD)

Note that full information on the approval of the study protocol must also be provided in the manuscript.

Flow Cytometry

Plots

Confirm that:

- [x] The axis labels state the marker and fluorochrome used (e.g. CD4-FITC).
- [x] The axis scales are clearly visible. Include numbers along axes only for bottom left plot of group (a 'group' is an analysis of identical markers).
- [x] All plots are contour plots with outliers or pseudocolor plots.
- [x] A numerical value for number of cells or percentage (with statistics) is provided.

Methodology

Sample preparation	Leukocyte single cell suspensions were prepared as decribed in methods section.
Instrument	LSR Fortessa and FACSCanto flow cytometer (Becton Dickinson)
Software	BD FacsDIVA Software version 6.1.3. and 6.2
Cell population abundance	*Describe the abundance of the relevant cell populations within post-sort fractions, providing details on the purity of the samples and how it was determined.*
Gating strategy	All gating strategies are described in figure legends, methods or text.

☐ Tick this box to confirm that a figure exemplifying the gating strategy is provided in the Supplementary Information.
